# Application of the Two-Dimensional Entropy Measures in the Infrared Thermography-Based Detection of Rider: Horse Bodyweight Ratio in Horseback Riding

**DOI:** 10.3390/s22166052

**Published:** 2022-08-13

**Authors:** Małgorzata Domino, Marta Borowska, Łukasz Zdrojkowski, Tomasz Jasiński, Urszula Sikorska, Michał Skibniewski, Małgorzata Maśko

**Affiliations:** 1 Department of Large Animal Diseases and Clinic, Institute of Veterinary Medicine, Warsaw University of Life Sciences, 02-787 Warsaw, Poland; lukasz_zdrojkowski@sggw.edu.pl (Ł.Z.); tomasz_jasinski@sggw.edu.pl (T.J.); 2 Institute of Biomedical Engineering, Faculty of Mechanical Engineering, Białystok University of Technology, 15-351 Bialystok, Poland; m.borowska@pb.edu.pl; 3 Department of Animal Breeding, Institute of Animal Science, Warsaw University of Life Sciences, 02-787 Warsaw, Poland; s193600@sggw.edu.pl; 4 Department of Morphological Sciences, Institute of Veterinary Medicine, Warsaw University of Life Sciences, 02-776 Warsaw, Poland; michal_skibniewski@sggw.edu.pl

**Keywords:** infrared thermography, texture analysis, entropy-based approaches, two-dimensional entropy, equine application

## Abstract

As obesity is a serious problem in the human population, overloading of the horse’s thoracolumbar region often affects sport and school horses. The advances in using infrared thermography (IRT) to assess the horse’s back overload will shortly integrate the IRT-based rider-horse fit into everyday equine practice. This study aimed to evaluate the applicability of entropy measures to select the most informative measures and color components, and the accuracy of rider:horse bodyweight ratio detection. Twelve horses were ridden by each of the six riders assigned to the light, moderate, and heavy groups. Thermal images were taken pre- and post-exercise. For each thermal image, two-dimensional sample (SampEn), fuzzy (FuzzEn), permutation (PermEn), dispersion (DispEn), and distribution (DistEn) entropies were measured in the withers and the thoracic spine areas. Among 40 returned measures, 30 entropy measures were exercise-dependent, whereas 8 entropy measures were bodyweight ratio-dependent. Moreover, three entropy measures demonstrated similarities to entropy-related gray level co-occurrence matrix (GLCM) texture features, confirming the higher irregularity and complexity of thermal image texture when horses worked under heavy riders. An application of DispEn to red color components enables identification of the light and heavy rider groups with higher accuracy than the previously used entropy-related GLCM texture features.

## 1. Introduction

Obesity is a serious health [1] and societal problem [2] that occurs worldwide on an increasing scale [3]. When the coronavirus disease 2019 (COVID-19) [4] was declared a pandemic by the World Health Organization (WHO), many governments decided to introduce lockdowns, as a part of interventions to diminish the spread of the virus [5]. COVID-19-related lockdown forced many people, including horse owners [6,7], horseracing staff [8], and equestrian athletes [9], to stay at home and limit their physical activity [10]. To a lesser extent for professional riders [9] and more for young horse riders [11] and leisure horse owners [6,7,12], lockdown increases the incidence of obesity and related health risks [13].

As the bodyweight of the horse riders increases [7,11,14], an increasing load negatively impacts the health of the horse’s back [15], physical exercise of the horse [16,17], and horse welfare [18]. Therefore, the appropriate fit of rider size to the horse size is becoming increasingly important in riding horse usage [19,20]. For this purpose, the rider:horse bodyweight ratio is used, as both the weight of the horse, the saddle, and the rider influence the mutual fit [17,20]. The rider:horse bodyweight ratio is the ratio of the bodyweight of the horse to the rider plus saddle weight, expressed as a percentage [17]. Depending on the research, the rider:horse bodyweight ratio reflects a fitting horse with a rider of normal weight, when it ranges from 10 to 12% [17,20], through 10.6% [21], to <15% [15]. The rider is overweight in relation to the horse’s size when the rider:horse bodyweight ranges from >12% ≤15% [17,20] to 15–20% [15]. Finally, the rider is obese in relation to the horse’s size when the rider:horse bodyweight ranges from >15% [17,20], through 21.3% [21], to 20–30% [15].

With the introduction of data processing [22,23,24,25,26], the development of technology for biometric measurements [24,25,26,27,28,29,30,31,32,33] and its introduction to equine medicine and care, there is a growing need to develop innovations that support the diagnosis of the rider:horse bodyweight ratio. According to the World Horse Welfare (WHW) and the British Equestrian Federation (BEF), riders should have the possibility to assess if they represent an appropriate bodyweight concerning their horse’s size; thus, every effort should be made to develop innovative assessment methods for the rider-horse fit [17]. The use of mathematical classification [22,23] and machine learning [24,25,26,27] methods constitute a new direction in the development of monitoring equine physiology [22,24] and the reaction of the horse’s organism to exercise loads [23,25] and diseases [26,27]. In the field of non-invasive horse monitoring, there is progress in the use of biometric devices, including inertial measurement units (IMU) to monitor motion speed [24], motion caption [28], and breath-jockey to assess the respiratory rate and kinematic parameters of performance horses [29], single inertial sensor (SIS) for the evaluation of the neuromusculoskeletal system of performance horses [30,31], and infrared thermography (IRT) for quantification of the radiant energy emitted by the body surface, which is proportional to the horse’s effort intensity [32,33]. Considering the advances made in recent years in the use of IRT to assess the rider:horse bodyweight ratio [20,21,34] and the rapidly increasing availability of smartphone thermal camera software and medical applications [35,36,37,38], the IRT-based assessment of rider-horse fit may be shortly integrated into everyday equine practice.

It has been recently demonstrated that conventional IRT allows the detection of differences in the horse’s superficial body temperature between riders that represent a 10.6% and 21.3% rider:horse bodyweight ratio [21]; however, this is not the case between riders with a 10.1% and 15.3% rider:horse bodyweight ratio [34]. Detection of the differences between riders that represent a 10.1% and 15.3% rider:horse bodyweight ratio was carried out using texture analysis thermal images based on the gray-level matrices (GLM) [34]. Extending the texture analysis to more areas in the thoracolumbar region and by conversion of thermal images to color components, it was shown that consistent measurable differences exist in texture features between thermal images taken from the thoracolumbar region of horses worked under riders with a 10–12%, >12 ≤15%, and >15 <18% rider:horse bodyweight ratio [20]. The differences were observed primarily in the red component of the IRT images and especially in texture heterogeneity measures, such as SumEntrp, Entropy, and DifEntrp [20]; therefore, in the current study relatively recently introduced entropy-based measurements [39] were used for quantitative description of the thermal images’ texture. In entropy-related gray-level matrices’ texture features, the entropy measures are calculated as a measured, disordered matrix obtained from the processing step applied to the image [40], whereas entropy-based measurements represent a new class of easy-to-implement methods [41], computed directly on the image [39,42] and related to image irregularity or complexity [39]. Therefore, the use of entropy-based measurements in equine thermal image analysis is the next necessary step in the successive development of technology, which in the long run may improve welfare outcomes for the horses used in equestrian activities. As entropy-based approaches need to be evaluated for usefulness in different types of applications [43], the preliminary and current studies introduce this method to equine practice. In the preliminary study, the entropy-based measurements of thermal image texture analysis have already been performed successfully in an equine model of pregnancy [44]. In the current study, five entropy-based measurements, including two-dimensional sample entropy [45], two-dimensional fuzzy entropy [46], two-dimensional permutation entropy [47], two-dimensional dispersion entropy [48], and two-dimensional distribution entropy [49], were used to find differences between thermal images of horses worked under riders with a 10–12%, >12 ≤15%, and >15 <18% rider:horse bodyweight ratio.

The current study aimed to transform thermal images obtained from the equine thoracolumbar region among riders that represent different rider:horse bodyweight ratios into four color components, segment two informative regions of interest, and extract measures of five entropy-based measurements. Then, the applicability of entropy measures for each color component was evaluated using specific criteria to select the most informative combinations. The selected entropy measures and color components were compared with recently reported entropy-related gray-level matrices texture features to find similarities. Finally, for the selected measures and features, the accuracy of rider:horse bodyweight ratio detection was calculated.

## 2. Materials and Methods

### 2.1. Horses and Riders

Twelve Polish warmblood, WULS-owned horses participated in the study. Horses (six geldings and six mares) were aged (mean ± SD) 9.3 ± 1.8 years old, with body weights of (mean ± SD) 566.7 ± 13.7 kg, and heights at the withers of (mean ± SD) 160.3 ± 3.9 cm. An appropriate saddle was fitted to each horse based on Greve and Dyson’s protocol [50]. Saddles weighed (mean; range) 4.3 kg; 4.1–4.5 kg. Six female riders, members of the Animal Sciences Students Riding Association (ASSRA) in WULS, participated in the study. Riders were aged (mean ± SD) 30.5 ± 7.1 years old, with body weights of (mean ± SD) 75.5 ± 14.6 kg, and heights of (mean ± SD) 166.8 ± 6.6 cm. Riders were divided into the following three rider groups: L, light; M, moderate; H, heavy, based on the rider’s bodyweight and rider’s body mass index (BMI). The inclusion criteria to the light rider groups were bodyweight (mean; range) 59.0 kg; 58.0–60.0 kg and BMI (mean; range) 23.2 kg/m^2^; 22.9–23.4 kg/m^2^. The inclusion criteria to the moderate rider groups were bodyweight (mean; range) 76.0 kg; 77.0–75.0 kg and BMI (mean; range) 27.0 kg/m^2^; 26.0–27.9 kg/m^2^. The inclusion criteria to the heavy rider groups were bodyweight (mean; range) 91.5 kg; 91.0–92.0 kg and BMI (mean; range) 30.6 kg/m^2^; 30.8–30.4 kg/m^2^. The details of horses, saddles, and riders were presented in Supplementary Table S1, which is available online. The details of horses, saddles, and riders were used to calculate the rider:horse bodyweight ratio expressed as a percentage, based on Dyson et al.’s [17] protocol. The inclusion criterion for light rider groups was the rider:horse bodyweight ratios (mean; range) 11.2%; 10.6–11.8%. The inclusion criterion for moderate rider groups was the rider:horse bodyweight ratios (mean; range) 14.2%; 13.5–14.9%. In addition, the inclusion criterion for heavy rider groups was the rider:horse bodyweight ratios (mean; range) 16.9%; 16.3–17.7%. The details of rider:horse bodyweight ratio were presented in Supplementary Table S2, which is available online.

### 2.2. Study Protocol

**Figure 1:**
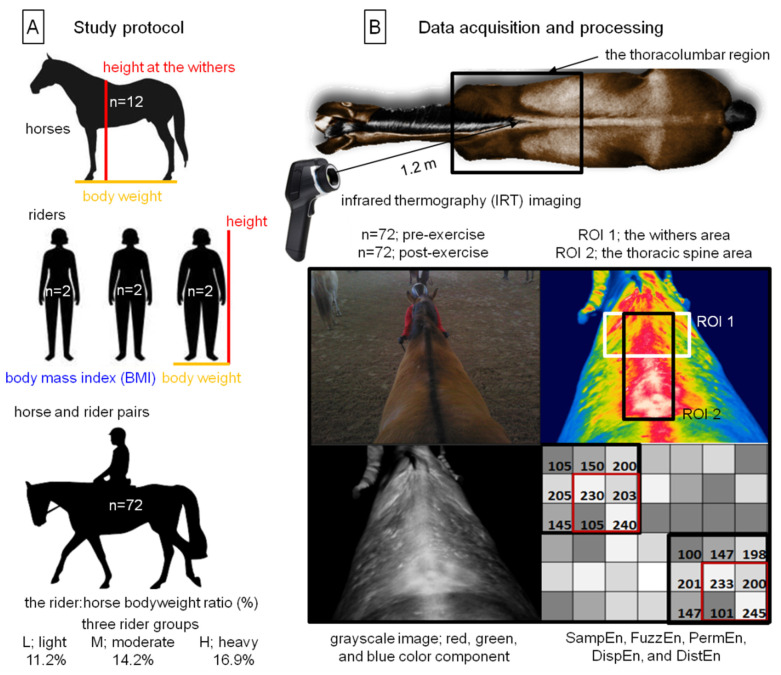
Schema of the material and methods used in the study. (**A**) The study protocol included horses (n = 12); riders (n = 6); and horse and rider pairs (n = 72), representing three rider groups that differed depending on the rider:horse bodyweight. (**B**) Data acquisition and processing included pre- and post-exercise infrared thermography (IRT) imaging of the thoracolumbar region; segmentation of two regions of interest (ROIs); thermal images converted into the grayscale images and three color components (red, green, blue); and extraction of the following five entropy measures: two-dimensional sample entropy (SampEn), two-dimensional fuzzy entropy (FuzzEn), two-dimensional permutation entropy (PermEn), two-dimensional dispersion entropy (DispEn), and two-dimensional distribution entropy (DistEn).

The study protocol was approved by the II Local Ethical Committee on Animal Testing in Warsaw on behalf of the National Ethical Committees on Animal Testing (No WAW2/034/2018, 27 April 2018) (Figure 1A).

#### 2.2.1. Preparation for Data Acquisition

Prior to the study, horses were in daily leisure use (leisure riding for up to 2 h per day, 6 days a week) in the Didactic Stable of Horse Breeding Division WULS. Prior to and during the study, horses were housed in individual stalls. The same management was provided for each horse, including individually calculated feeding (three rations per day of hay, oats, and concentrate fitting to horses’ nutritional requirements) and free access to fresh water and mineral salt block. During the study, horses were clinically healthy. The health status of the horses was confirmed based on the results of a basic clinical examination (heart rate, respiratory rate, capillary refill time, rectal temperature, mucous membranes, and lymph nodes examination) [51]; detailed back examination (the presence of tension in the muscles, lumps, abnormal hair wear, and reaction to pain examination) [51], and detailed orthopedic examination (the lameness examination) [52]. One week before the start of the study, horses were measured at the withers with a standard equine measure (Busse Sportartikel GmbH & Co. KG, Lohne, Germany) and weighed using equine platform weights (Baka-Wag, Bydgoszcz, Poland). One week before the start of the study, all saddles were fitted to each horse and weighed using personal weights (Soehnle, Nassau, Germany). On the study day, all riders were measured with a standard equine measure (Busse Sportartikel GmbH & Co. KG, Lohne, Germany) and weighed using personal weights (Soehnle, Nassau, Germany). In addition, 30 min before data acquisition, the thoracolumbar region of the horse’s back was brushed to remove dirt and mud and the horses were led to an indoor riding arena to acclimatize to the environmental conditions.

#### 2.2.2. Data Acquisition

Data in the form of IRT images were acquired over a 6-day test period. Each of the six horses was ridden once every day, by different studied riders. Each of the twelve riders rode two horses every day. All pairs (horse and rider) underwent the same standardized exercise test [17] in a familiar indoor riding arena (20 × 60 m), directly connected to the horse’s stable. In the indoor riding arena, the ambient temperature and relative humidity were in the range of 20.1 ± 0.9 °C and 50.5 ± 2.8% during the 6-day test period. All pairs were IRT imaged twice, before and after the standardized exercise test. Images before exercise were taken before saddling. Then, the horses were saddled and underwent a standardized exercise test. Images after effort were taken immediately after unsaddling. Thus, a total of 144 thermal images were obtained, of which 72 thermal images were annotated as pre-exercise and 72 as post-exercise, respectively. All pairs were IRT imaged using a non-contact thermographic camera (FLIR Therma CAM E25, FLIR Systems Brasil, Sorocaba, Brazil; emissivity (e) 0.99; temperature range between 10.0 and 50.0 °C) from the distance approximately 1.2 m, following the standardized previously described protocol [34]. All thermal images were taken by the same researcher (M.M.) (Figure 1B).

### 2.3. Data Processing

The obtained thermal images were manually segmented, and two regions of interest (ROIs) were annotated. The first ROI represented the withers area (ROI 1), whereas the second ROI represented the thoracic spine area (ROI 2) (Figure 1B). The annotated thermal images were converted to bitmaps and the based entropy image texture analysis was performed using the ImageJ software (Wayne Rasband, National Institutes of Mental Health, Bethesda, MD, USA). Each ROI was converted into its grayscale and three color components (red, green, blue), using four image conversion methods in Python version 3.8.5 64-bit [44], as well as the packages NumPy (https://numpy.org/ accessed on 9 May 2022), OpenCV (https://pypi.org/project/opencv-python/ accessed on 9 May 2022), and scikit-image [53]. In each color component, the entropy-based texture analysis was performed and five entropy measures were extracted using Python, version 3.8.5 64-bit using package EntopyHub [54], separately. The following entropy measures were considered: two-dimensional sample entropy (SampEn), two-dimensional fuzzy entropy (FuzzEn), two-dimensional permutation entropy (PermEn), two-dimensional dispersion entropy (DispEn), and two-dimensional distribution entropy (DistEn).

#### 2.3.1. Two-Dimensional Sample Entropy

The two-dimensional sample entropy (SampEn) was used to measure the irregularity in the pixel patterns [45].

SampEn determines the probability of similarity of patterns of length m with patterns of length *m* + 1. The similarity is calculated by comparing corresponding pixels in windows of length *m*. If a pixel in one window differs no more than *r* from the corresponding pixel in the other window, it means that the patterns are similar. The probability is obtained by calculating the total number of matches of patterns of length m and *m* + 1 according to the following formulas:

Φm(r)=1Nm∑j=1,j≠iN−m(1Nm−1∑a=1,b=1,(a,b)≠(i,j)N−m[#of xm(i,j)|d[xm(i,j),xm(a,b)]≤r])

Φm+1(r)=1Nm∑i,j=1,j≠iN−m(1Nm−1∑a=1,b=1,(a,b)≠(i,j)N−m[#of xm+1(i,j)|d[xm+1(i,j),xm+1(a,b)]≤r])

where d[·] is the distance function between neighboring windows $x_{m}\left(i,j\right)$ and $x_{m}\left(a,b\right)$ , defined as the maximum absolute difference of the correspondence scalar components and $N_{m}$ is the total number of square windows of the image *x*.

The ratio of $\Phi^{m}$ and $\Phi^{m+1}$ gives the conditional probability of finding similar patterns of length *m* + 1, given that they are similar for *m* [45,55]. SampEn is defined as

$$\mathrm{SampEn}=-\ln\frac{\Phi^{m+1}}{\Phi^{m}}\;$$

The regular images or periodic structures in the images have the same number of patterns for both m and *m* + 1, so a low entropy value is obtained. On the other hand, high entropy values are achieved for irregular images [45,56].

#### 2.3.2. Two-Dimensional Fuzzy Entropy

Two-dimensional fuzzy entropy is also used to quantify the irregularity in pixel patterns but uses a continuous exponential function to determine the degree of similarity [46].

Two-dimensional fuzzy entropy determines the probability of similarity by comparison patterns for their corresponding m points with *m* + 1 points according to the following formulas:

$$\Phi^{m}\left(r\right)=\frac{1}{N_{m}}\overset{N-m}{\underset{i=1,j=1}{\sum }}\left(\frac{1}{N_{m}-1}\overset{N-m}{\underset{a=1,b=1,\left(a,b\right)\neq \left(i,j\right)}{\sum }}D_{m}\left(i,j,a,b\right)\right)$$

$$\Phi^{m+1}\left(r\right)=\frac{1}{N_{m}}\overset{N-m}{\underset{i=1,j=1}{\sum }}\left(\frac{1}{N_{m}-1}\overset{N-m}{\underset{a=1,b=1,\left(a,b\right)\neq \left(i,j\right)}{\sum }}D_{m+1}\left(i,j,a,b\right)\right)$$

where $D_{m}$ is a continuous exponential function, defined as

Dm(i,j,a,b)=exp(−dm(i,j,a,b)2r)

d[·] is the distance function defined as the maximum absolute difference of the corresponding scalar components; *r* is the width of the boundary of the exponential function. FuzzyEn is defined as the negative natural logarithm of the conditional probability, shown by the following equation:

$$\mathrm{FuzzyEn}=-\ln\frac{\Phi^{m+1}\left(r\right)}{\Phi^{m}\left(r\right)}$$

Images with regular patterns or periodic structures achieve low FuzzyEn values, while images with irregular patterns or non-periodic structures reach high FuzzyEn value [46,57].

#### 2.3.3. Two-Dimensional Permutation Entropy

Two-dimensional permutation entropy is used to identify the irregular structure of the image [47].

PermEn is based on the concept of counting permutation patterns π . The permutation patterns are obtained after ordering the positions of the initial image patterns. The PermEn algorithm calculates the number of patterns that exist in the image for each permutation pattern [47]. The probability of each pattern is defined as

$$P=\left\{p\left(\pi \right);\pi =1,\ldots ,\left(d_{n}\times d_{m}\right)!\right\}$$

where $d_{n}$ and $d_{m}$ determine the number of accessible states of permutations.

PermEn is defined as

$$\mathrm{PermEn}=-\frac{1}{\left(n-d_{n}+1\right)\left(m-d_{m}+1\right)}\overset{d_{n}!\times d_{m}!}{\underset{\pi =1}{\sum }}p\left(\pi \right)\ln p\left(\pi \right)$$

If the image pixels are highly disordered, the permutation entropy value is close to one. In contrast, the PermEn value is close to zero if the pixels always appear in the same order [42,58].

#### 2.3.4. Two-Dimensional Dispersion Entropy

Two-dimensional dispersion entropy is used similarly to SampEn to assess the regularity of images, eliminating its limitations of indeterminacy of small-sized images and being computationally expensive for real-world applications [48].

Two-dimensional dispersion entropy relies on mapped to c classes and the values of image pixels form $z_{i,j\;}^{c}\;round\left(c\times v\left(i,j\right)+0.5\right)$ , where v(i,j) uses the sigmoid function, which is defined as:

$$v\left(i,j\right)=\frac{1}{\sigma \sqrt{2\pi }}\int_{-\infty }^{x\left(i,j\right)}e^{\frac{-{\left(t-\mu \right)}^{2}}{2\sigma^{2}}}dt$$

μ and σ represent the mean and the standard deviation of the original image.

After mapping, the obtained results are matched to the dispersion pattern $\pi_{v}$ and the probabilities $p\left(\pi_{v}\right)$ of each dispersion patterns are calculated [48] as

$$P=\left\{p\left(\pi_{v}\right);\pi_{v}=1,\ldots ,\left(d_{n}\times d_{m}\right)!\right\}$$

Finally, DispEn is defined as

$$\mathrm{DispEn}=-\frac{1}{\left(n-d_{n}+1\right)\left(m-d_{m}+1\right)}\overset{d_{n}!\times d_{m}!}{\underset{\pi =1}{\sum }}p\left(\pi_{v}\right)\ln p\left(\pi_{v}\right)$$

If all possible two-dimensional image dispersion patterns have the same probability value, DispEn reaches its maximum value. If there is one probability value p that is other than zero, DispEn reaches the minimum value and the image has a regular pattern [48,57].

#### 2.3.5. Two-Dimensional Distribution Entropy

Two-dimensional distribution entropy is used for the quantitative description of the irregularities of the images, taking into account the small size of the image [49].

Two-dimensional distribution entropy, in the process of counting the amount of similarity between two windows, measures the distance between the corresponding windows. The histogram of the distance matrix is used to estimate the empirical probability density function (ePDF) [49,59].

Finally, DistEn is defined as

$$\mathrm{DistEn}=-\overset{M}{\underset{t=1}{\sum }}p_{t}log_{2}\left(p_{t}\right)$$

where $p_{t}$ is probability of each histogram bin, *M* is the number of bins.

#### 2.3.6. Entropy-Related Gray Level Co-Occurrence Matrix Texture Features

The entropy-related gray level co-occurrence matrix texture features are used to evaluate randomness in the image or in neighbourhood intensity value differences [60].

In the same ROIs and color components, the thirty-one texture features in three analytical approaches, including histogram statistics (HS; 13 features), gray-level run-length matrix (GLRLM; 7 features), and gray level co-occurrence matrix (GLCM; 11 features), were returned using the QMazda Software (http://www.eletel.p.lodz.pl/pms/SoftwareQmazda.html accessed on 9 May 2022) [61]. These results have been previously reported [20]. In Domino et al. [20], three entropy-related GLCM texture features in the red component of IRT images, SumEntrp, Entropy, and DifEntrp, were considered as consistent measurable differences between IRT taken from the equine thoracolumbar region among riders that represent different rider:horse bodyweight ratios. The raw data of SumEntrp, Entropy, and DifEntrp in ROI 1 and 2 from a previous study [20] were used in the present study to find similarities between the selected entropy measures and the selected entropy-related GLCM texture features.

### 2.4. Statistical Analysis

Statistical analysis was performed using GraphPad Prism6 software (GraphPad Software Inc., San Diego, CA, USA). Data from five entropy measures (SampEn, FuzzEn, PermEn, DispEn, DistEn) were presented as data series for grayscale images and three color components (red, green, blue) independently, where each horse represented one realization. The numerical data in Supplementary Tables S3–S10 were presented as mean ± standard deviation (SD). Data series were tested independently for univariate distributions using a Shapiro–Wilk normality test.

The comparisons between the pre-exercise and post-exercise data series were assessed using the paired *t*-test for Gaussian data and the Wilcoxon matched-pairs signed-rank test for non-Gaussian data. The alpha value was established as α = 0.05. Those measures that differed between the pre-exercise and post-exercise imaging were marked by color (for L group—light gray, red, green, blue; for M group—moderate gray, red, green, blue; and for H group—dark gray, red, green, blue) and by a cross (X) in the appropriate figure. Only those measures that differed between the pre-exercise and post-exercise imaging for all three groups (L, M, H), simultaneously, were selected for further analysis.

The comparisons between the light (L), moderate (M), and heavy (H) rider groups data series were assessed using the ordinary one-way ANOVA followed, by Tukey’s multiple comparisons test for Gaussian data and the Kruskal–Wallis test, followed by the Dunn’s multiple comparisons test, for non-Gaussian data. The alpha value was established as α = 0.05. The minimum and maximum values, lower and upper quartiles, and median were used for data presentation. On box plots, the cross indicates the mean value, and each realization is displayed. Only those measures that differed between the rider groups were marked in the appropriate figure (by colors: red, green, blue, and a cross (X)). Only those measures were selected for further analysis.

For the selected entropy measures and the three selected entropy-related GLCM texture features, the linear regressions were calculated. On regression plots, two regression equations were displayed, including one for each selected entropy measure and one for SumEntrp, Entropy, or DifEntrp. Equations were supported with the measure of the difference of linearity. All the slopes were significantly non-zero (*p* < 0.0001). For no significant difference between the slopes (*p* > 0.05), one slope was calculated, and the intercepts were compared. For no significant differences between the intercepts (*p* > 0.05), one intercept was calculated. On regression plots, when the slopes did not differ between the measures (entropy measures and entropy-related GLCM texture features), the plot was marked (by solid frames and colors: red, green, or blue). When the slope value of the entropy measures was higher than the slope value of the entropy-related GLCM texture features, the plot was additionally marked (by dashed frames and colors: red, green, or blue). When the slope value of one or more of entropy-related GLCM texture features was lower than at least half of the slope value of entropy measures, the entropy-related GLCM texture feature was not considered for further analysis. Only those entropy measures for which the slopes were not significantly different compared to at least two selected entropy-related GLCM texture features were marked in the appropriate figure (by colors: red, green, blue, and a cross (X)). Only those measures were selected for further analysis.

The accuracy of rider:horse bodyweight ratio detection was calculated using three thresholds for gradually increasing measures (mean − SD (m − SD), mean, mean + SD (m + SD)). The distinguishing of light (L) and heavy (H) rider groups was considered. The image was annotated as a heavy rider (1) when the individual measure value was above the threshold and annotated as a light rider (0) when below it. The same annotation was carried out for the light and heavy rider groups. The sensitivity (Se), specificity (Sp), positive predictive value (PPV), and negative predictive value (NPV) of rider:horse bodyweight ratio detection were estimated. The values of Se, Sp, PPV, and NPV were calculated across the range from 0.1 to 1.0 using standard formulae [62].

## 3. Results

**Figure 2:**
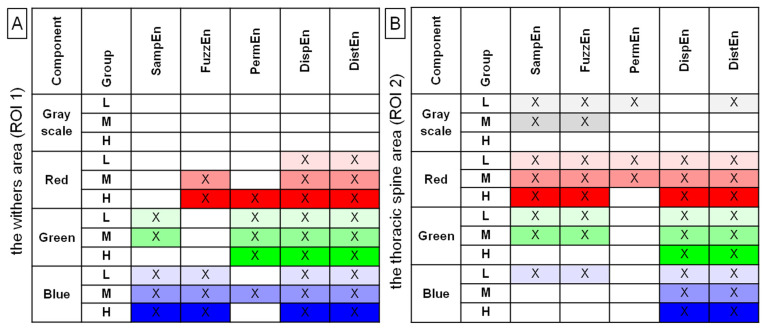
Entropy measures for examined grayscale images (grayscale) and color components (red, green, blue), which were found to be significantly different between the pre-exercise and post-exercise imaging for all rider groups (L, light; M, moderate; H, heavy). Data were presented separately for the withers area (ROI 1) (**A**) and the thoracic spine area (ROI 2) (**B**). SampEn—two-dimensional sample entropy, FuzzEn—two-dimensional fuzzy entropy, PermEn—two-dimensional permutation entropy, DispEn—two-dimensional dispersion entropy, DistEn—two-dimensional distribution entropy. The measures that differed between the pre-exercise and post-exercise imaging were marked by color (light gray, red, green, blue for L group; moderate gray, red, green, blue for M group; and dark gray, red, green, blue for H group) and by a cross (X).

Among 40 returned combinations of entropy measures (n = 5) and color components (n = 4) calculated in both ROIs (n = 2), 4 entropy measures for grayscale images, 9 entropy measures for red color components, 8 entropy measures for green color components, and 9 entropy measures for blue color components differed significantly between the pre-exercise and post-exercise imaging for at least 1 of the rider groups. These combinations were summarized in Figure 2.

When those entropy measures that differed simultaneously between the pre-exercise and post-exercise imaging for all three rider groups were considered, 17 combinations passed the selection criterion, including none for grayscale images, 5 for green color components, and each 6 for both red and blue color components. Therefore, in ROI 1, DispEn and DistEn for red color components; PermEn, DispEn, and DistEn for green color components; and SampEn, FuzzEn, DispEn, and DistEn for blue color components were considered for further analysis. In ROI 2, SampEn, FuzzEn, DispEn, and DistEn for red color components; DispEn and DistEn for green color components; and DispEn and DistEn for blue color components were considered for further analysis. The details of this initial comparison, as well as the values (mean ± SD) of all examined entropy measures, were presented in Supplementary Tables S3–S10, which are available online.

**Figure 3:**
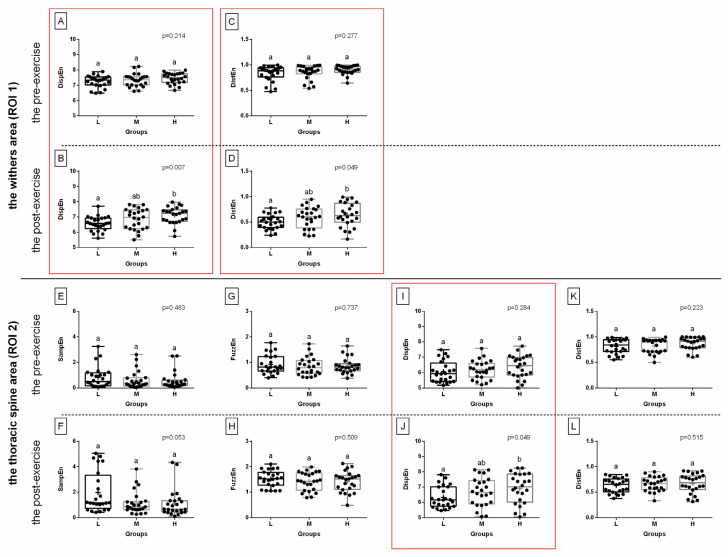
The comparison of the selected entropy measures in the red color component between light (L), moderate (M), and heavy (H) rider groups. The withers area (ROI 1) (**A**–**D**) and the thoracic spine area (ROI 2) (**E**–**L**) are separated by a solid horizontal line. The images obtained pre-exercise (**A**,**C**,**E**,**G**,**I**,**K**) and post-exercise (**B**,**D**,**F**,**H**,**J**,**L**) are separated by dashed horizontal lines. The following entropy measures are considered: DispEn—two-dimensional dispersion entropy (**A**,**B**,**I**,**J**), DistEn—two-dimensional distribution entropy (**C**,**D**,**K**,**L**), SampEn—two-dimensional sample entropy (**E**,**F**), FuzzEn—two-dimensional fuzzy entropy (**G**,**H**). Data are presented using minimum and maximum values, lower and upper quartiles, and median. The mean value is marked by a cross. Differences between rider groups were indicated with individual *p*-values when *p* < 0.05. Different superscripts on each plot were statistically different. Measures that differ between rider groups (L, M, H) are marked with a red frame.

**Figure 4:**
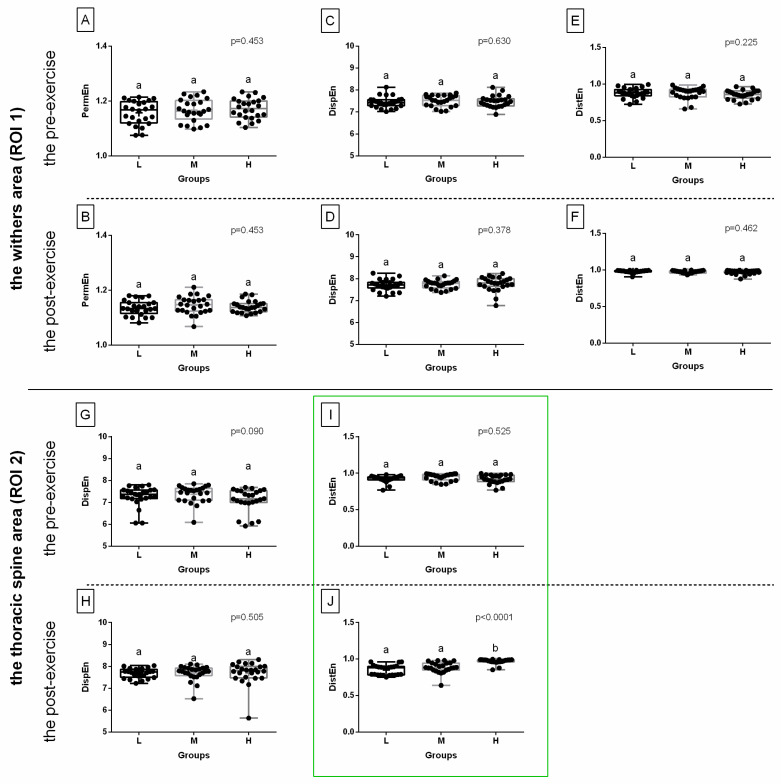
The comparison of the selected entropy measures in the green color component between light (L), moderate (M), and heavy (H) rider groups. The withers area (ROI 1) (**A**–**F**) and the thoracic spine area (ROI 2) (**G**–**J**) are separated by a solid horizontal line. The images obtained pre-exercise (**A**,**C**,**E**,**G**,**I**) and post-exercise (**B**,**D**,**F**,**H**,**J**) are separated by dashed horizontal lines. The following entropy measures are considered: PermEn—two-dimensional permutation entropy (**A**,**B**), DispEn—two-dimensional dispersion entropy (**C**,**D**,**G**,**H**), DistEn—two-dimensional distribution entropy (**E**,**F**,**I**,**J**). Data are presented using minimum and maximum values, lower and upper quartiles, and median. The mean value is marked by a cross. Differences between rider groups were indicated with individual *p*-values when *p* < 0.05. Different superscripts on each plot were statistically different. Measures that differ between rider groups (L, M, H) are marked with a green frame.

**Figure 5:**
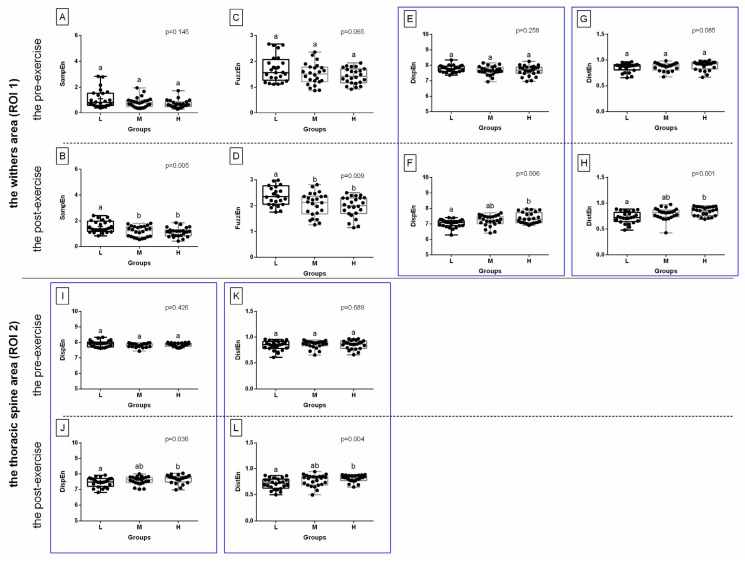
The comparison of the selected entropy measures in the blue color component between light (L), moderate (M), and heavy (H) rider groups. The withers area (ROI 1) (**A**–**H**) and the thoracic spine area (ROI 2) (**I**–**L**) are separated by a solid horizontal line. The images obtained pre-exercise (**A**,**C**,**E**,**G**,**I**,**K**) and post-exercise (**B**,**D**,**F**,**H**,**J**,**L**) are separated by dashed horizontal lines. The following entropy measures are considered: SampEn—two-dimensional sample entropy (**A**,**B**), FuzzEn—two-dimensional fuzzy entropy (**C**,**D**), DispEn—two-dimensional dispersion entropy (**E**,**F**,**I**,**J**), DistEn—two-dimensional distribution entropy (**G**,**H**,**K**,**L**). Data are presented using minimum and maximum values, lower and upper quartiles, and median. The mean value is marked by a cross. Differences between rider groups were indicated with individual *p*-values when *p* < 0.05. Different superscripts on each plot were statistically different. Measures that differ between rider groups (L, M, H) are marked with a blue frame.

When comparing selected post-exercise entropy measures between rider groups (L, light; M, moderate; H, heavy), no difference was found in ROI 1 for three entropy measures for green color components, or for two entropy measures for blue color components; this was also the case in ROI 2 for three entropy measures for red color components and one entropy measure for green color components (Figure 3, Figure 4 and Figure 5). However, rider:horse bodyweight ratio-dependent differences were observed in ROI 1 for two entropy measures for red color components and two entropy measures for blue color components; as well as in ROI 2 for one entropy measure for red color components, one entropy measure for green color components, and two entropy measures for blue color components (Figure 3, Figure 4 and Figure 5).

For red color component post-exercise measures, in ROI 1, DispEn and DistEn were higher in the heavy rider group than in the light rider group, with no differences between the heavy and light rider groups or moderate rider group. However, in ROI 2, similar differences were observed only for DispEn (Figure 3).

For green color component post-exercise measures, in ROI 2 only, DistEn was higher in the heavy rider group than in the light and moderate rider groups, with no differences between the light and moderate rider groups (Figure 4).

**Figure 6:**
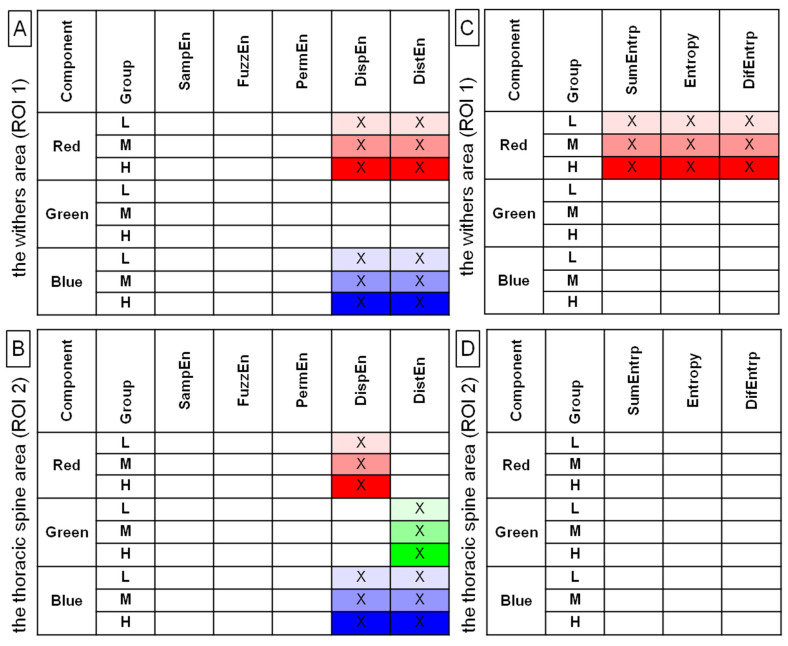
Entropy measures (**A**,**B**) and selected gray-level run-length matrix features (**C**,**D**) for examined color components (red, green, blue), which were found to be significantly different between light (L), moderate (M), and heavy (H) rider groups on the post-exercise imaging. Data were presented separately for the withers area (ROI 1) (**A**,**C**) and the thoracic spine area (ROI 2) (**B**,**D**). SampEn—two-dimensional sample entropy, FuzzEn—two-dimensional fuzzy entropy, PermEn—two-dimensional permutation entropy, DispEn—two-dimensional dispersion entropy, DistEn—two-dimensional distribution entropy, SumEntrp—summation entropy, entropy, DifEntrp—differential entropy. The measures that differed between rider groups were marked by color (light red, green, blue for L group; moderate red, green, blue for M group; and dark red, green, blue for H group) and by a cross (X).

For blue color component post-exercise measures, both in ROI 1 and ROI 2, DispEn and DistEn were higher in the heavy rider group than in the light rider group, with no differences between the heavy and light rider groups or moderate rider group (Figure 5). Only those entropy measures that differed between the rider groups were considered for further analysis and are summarized in Figure 6A,B. In Figure 6C,D, the SumEntrp, Entropy, and DifEntrp from a previous study [20] were introduced to find similarities between the selected entropy measures and the selected entropy-related GLCM texture features. The SumEntrp, Entropy, and DifEntrp differed between the rider groups only for the red color component in ROI 1 (Figure 6C).

**Figure 7:**
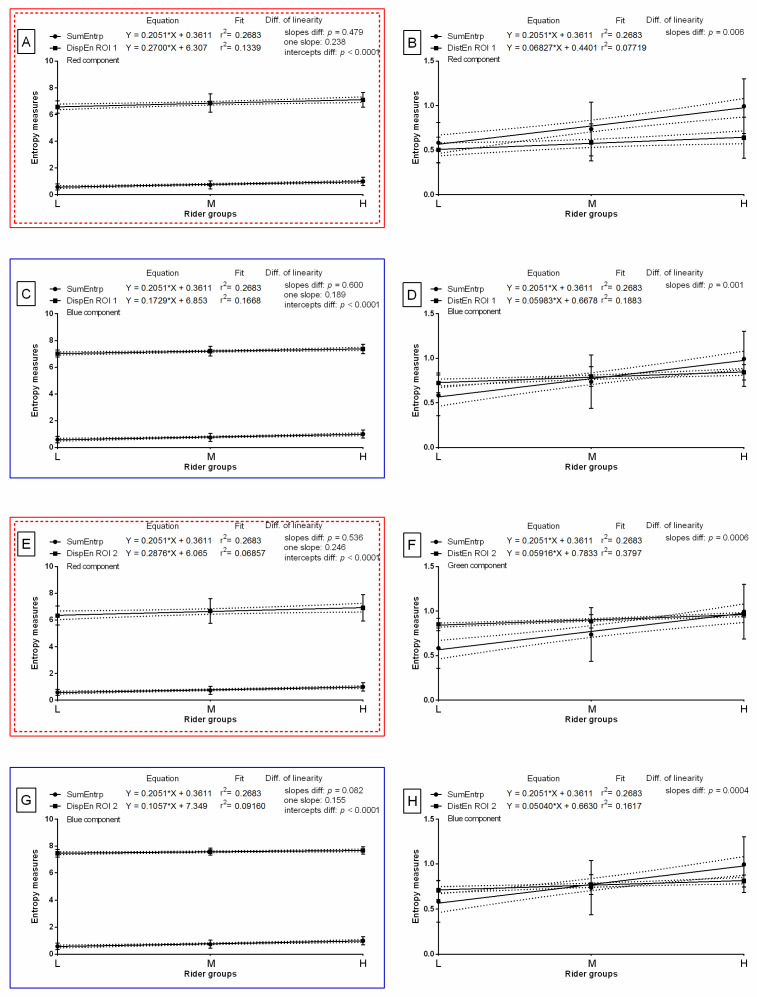
Comparison of studied entropy measures (DispEn—two-dimensional dispersion entropy, DistEn—two-dimensional distribution entropy) and selected gray-level run-length matrix feature (SumEntrp—summation entropy) for examined color components (red, (**A**,**B**,**E**); green, (**F**); blue, (**C**,**D**,**G**,**H**)), which were found to be significantly different between light (L), moderate (M), and heavy (H) rider groups on the post-exercise imaging. Data were presented separately for the withers area (ROI 1) (**A**–**D**) and the thoracic spine area (ROI 2) (**E**–**H**). Similarity was tested using linear regressions. A *p*-value of less than 0.05 was considered significant. If the difference between slopes was not significant, a single slope measurement was calculated. Plots with the entropy measures and slopes that did not differ with SumEntrp were marked by colored (red, blue) solid frames. Plots with the entropy measures and slope values that were higher than SumEntrp were marked by colored (red, blue) dashed frames.

The slope of the linear regression equations for SumEntrp compared to the slopes of the entropy measures were not significantly different and one slope measurement was calculated for DispEn for the red color component in ROI 1 (*p* = 0.479; one slope = 0.238; Figure 7A), DispEn for the blue color component in ROI 1 (*p* = 0.600; one slope = 0.189; Figure 7C), DispEn for the red color component in ROI 2 (*p* = 0.536; one slope = 0.246; Figure 7E), and DispEn for the blue color component in ROI 2 (*p* = 0.082 one slope = 0.155; Figure 7G). The intercepts within those data pairs were compared, and all differences between the intercepts were considered significant (*p* < 0.05). Therefore, one intercept was not calculated for each of the data pairs. For all the other compared data pairs, the slopes were significantly different (*p* < 0.05; Figure 7). The slope value of entropy measures was higher than the slope value of SumEntrp (slope = 0.205) for DispEn for the red color component in ROI 1 (slope = 0.270; Figure 7A) and for DispEn for the red color component in ROI 2 (slope = 0.288 Figure 7E).

**Figure 8:**
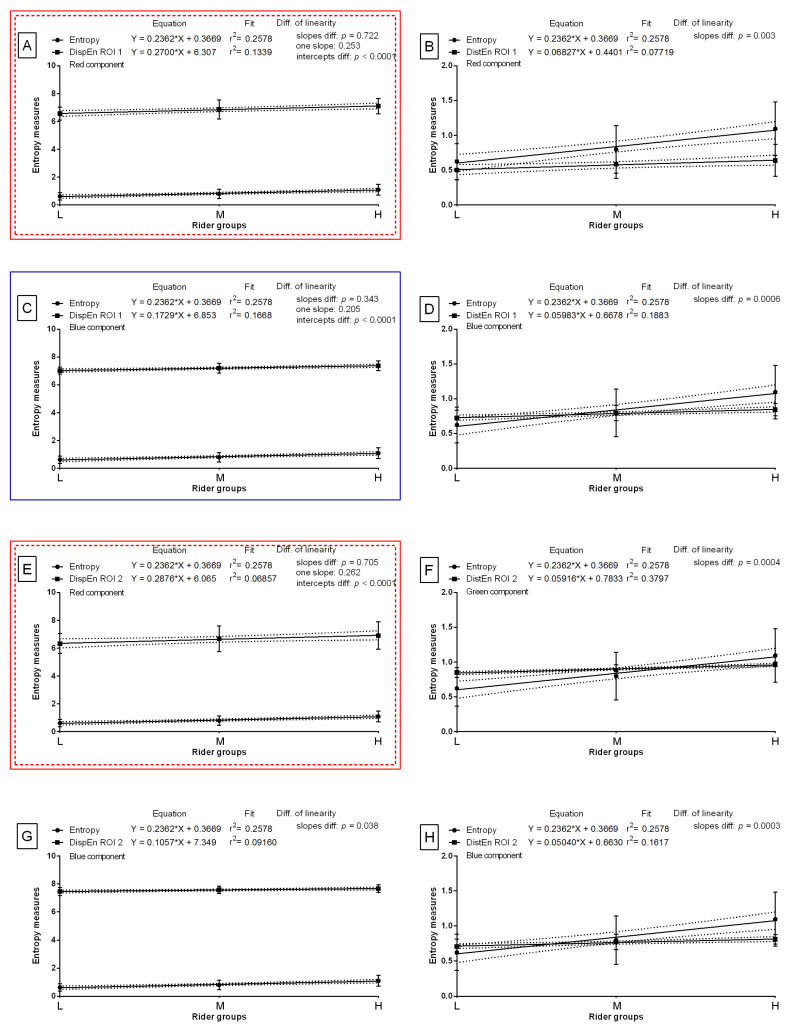
Comparison of studied entropy measures (DispEn—two-dimensional dispersion entropy, DistEn—two-dimensional distribution entropy) and selected gray-level run-length matrix feature (entropy) for examined color components (red, (**A**,**B**,**E**); green, (**F**); blue, (**C**,**D**,**G**,**H**)), which were found to be significantly different between light (L), moderate (M), and heavy (H) rider groups on the post-exercise imaging. Data were presented separately for the withers area (ROI 1) (**A**–**D**) and the thoracic spine area (ROI 2) (**E**–**H**). Similarity was tested using linear regressions. A *p*-value of less than 0.05 was considered significant. If the difference between slopes was not significant, a single slope measurement was calculated. Plots with the entropy measures and slopes that did not differ with entropy were marked by colored (red, blue) solid frames. Plots with the entropy measures and slope values that were higher than entropy were marked by colored (red, blue) dashed frames.

The slope of the linear regression equations for entropy compared to the slopes of the entropy measures were not significantly different and one slope measurement was calculated for DispEn for the red color component in ROI 1 (*p* = 0.722; one slope = 0.253; Figure 8A), DispEn for the blue color component in ROI 1 (*p* = 0.343; one slope = 0.205; Figure 8C), and DispEn for the red color component in ROI 2 (*p* = 0.705; one slope = 0.262; Figure 8E). The intercepts within those data pairs were compared, and all differences between the intercepts were considered significant (*p* < 0.05). Therefore, one intercept was not calculated for each of the data pairs. For all the other compared data pairs, the slopes were significantly different (*p* < 0.05; Figure 8). The slope value of entropy measures was higher than the slope value of entropy (slope = 0.236) for DispEn for the red color component in ROI 1 (slope = 0.270; Figure 8A) and for DispEn for the red color component in ROI 2 (slope = 0.288 Figure 8E).

**Figure 9:**
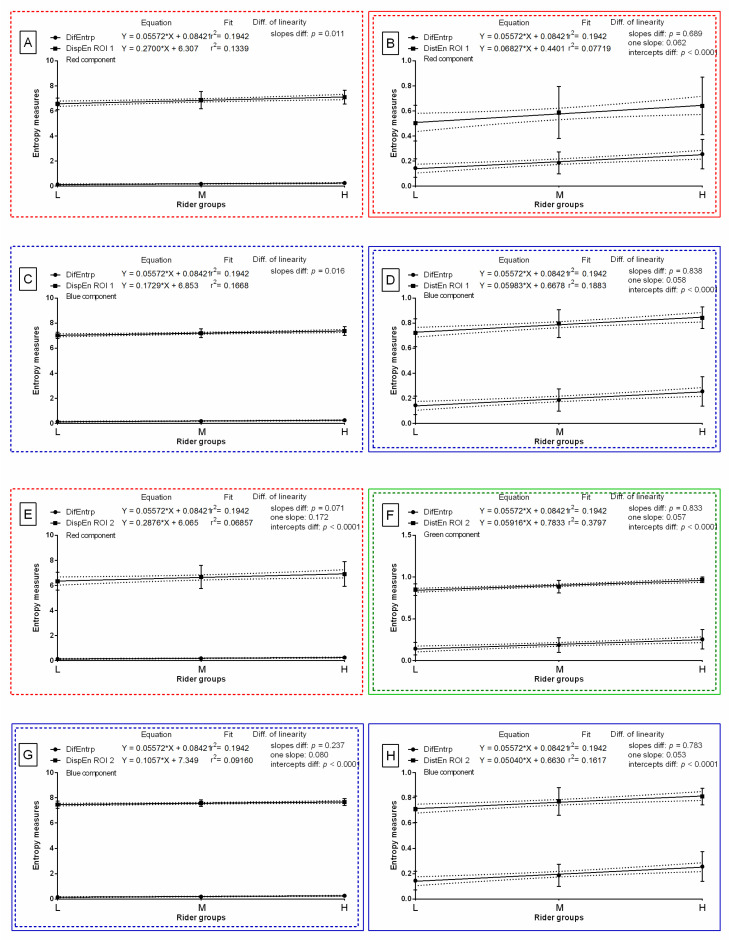
Comparison of studied entropy measures (DispEn—two-dimensional dispersion entropy, DistEn—two-dimensional distribution entropy) and selected gray-level run-length matrix feature (DifEntrp—differential entropy) for examined color components (red, (**A**,**B**,**E**); green, (**F**); blue, (**C**,**D**,**G**,**H**)), which were found to be significantly different between light (L), moderate (M), and heavy (H) rider groups on the post-exercise imaging. Data were presented separately for the withers area (ROI 1) (**A**–**D**) and the thoracic spine area (ROI 2) (**E**–**H**). Similarity was tested using linear regressions. A *p*-value of less than 0.05 was considered significant. If the difference between slopes was not significant, a single slope measurement was calculated. Plots with the entropy measures and slopes that did not differ with DifEntrp were marked by colored (red, green, blue) solid frame. Plots with the entropy measures and slope values that were higher than DifEntrp were marked by colored (red, green, blue) dashed frames.

The slope of the linear regression equations for DifEntrp compared to the slopes of the entropy measures were not significantly different and one slope measurement was calculated for DistEn for the red color component in ROI 1 (*p* = 0.689; one slope = 0.062; Figure 9B), DistEn for the blue color component in ROI 1 (*p* = 0.838; one slope = 0.058; Figure 9D), DistEn for the green color component in ROI 2 (*p* = 0.833; one slope = 0.057; Figure 9F), DispEn for the blue color component in ROI 2 (*p* = 0.237; one slope = 0.080; Figure 9G), and DistEn for the blue color component in ROI 2 (*p* = 0.783; one slope = 0.053; Figure 9H). The intercepts within those data pairs were compared, and all differences between the intercepts were considered significant (*p* < 0.05). Therefore, one intercept was not calculated for each of the data pairs. For all the other compared data pairs, the slopes were significantly different (*p* < 0.05; Figure 9). The slope value of entropy measures was higher than the slope value of DifEntrp (slope = 0.056) for DispEn for the red color component in ROI 1 (slope = 0.270; Figure 9A), DistEn for the red color component in ROI 1 (slope = 0.068; Figure 9B), DispEn for the blue color component in ROI 1 (slope = 0.173; Figure 9C), DistEn for the blue color component in ROI 1 (slope = 0.060; Figure 9D), DispEn for the red color component in ROI 2 (slope = 0.288 Figure 9E), DistEn for the green color component in ROI 2 (slope = 0.059; Figure 9F), and DispEn for the blue color component in ROI 2 (slope = 0.106; Figure 9G). The slope value of DifEntrp was lower than the slope value of seven out of eight selected entropy measures; thus, the DifEntrp was not considered for further analysis.

**Figure 10:**
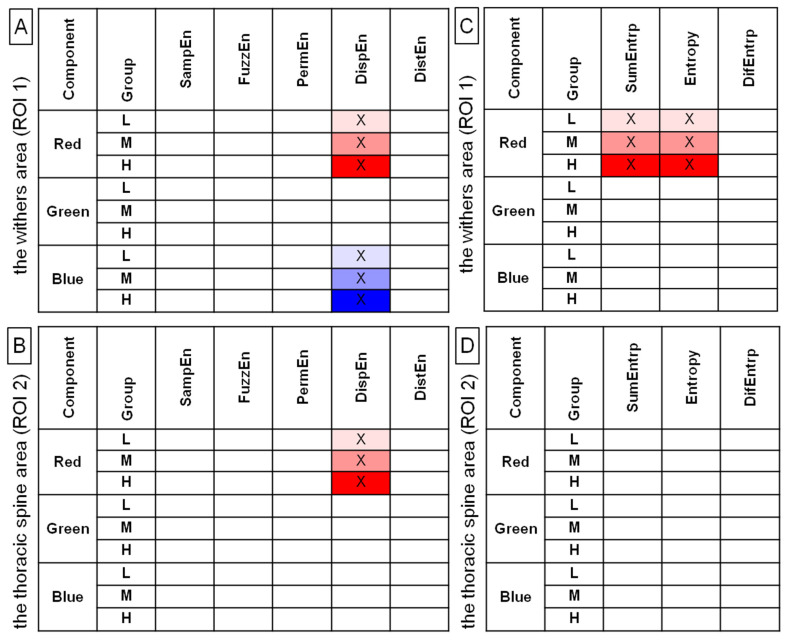
Entropy measures (**A**,**B**) and selected gray-level run-length matrix features (**C**,**D**) for examined color components (red, green, blue), which were considered to increase in a similar way throughout the light (L), moderate (M), and heavy (H) rider groups on the post-exercise imaging. Data were presented separately for the withers area (ROI 1) (**A**,**C**) and the thoracic spine area (ROI 2) (**B**,**D**). SampEn—two-dimensional sample entropy, FuzzEn—two-dimensional fuzzy entropy, PermEn—two-dimensional permutation entropy, DispEn—two-dimensional dispersion entropy, DistEn—two-dimensional distribution entropy, SumEntrp—summation entropy, entropy, DifEntrp—differential entropy. The similar measures were marked by color (light red, green, blue for L group; moderate red, green, blue for M group; and dark red, green, blue for H group) and by a cross (X).

Only those entropy measures that were considered to increase in a similar way to the selected entropy-related GLCM texture features throughout the light (L), moderate (M), and heavy (H) rider groups were considered for further analysis and are summarized in Figure 10. Therefore, the accuracy of rider:horse bodyweight ratio detection was calculated for DispEn for the red color component in ROI 1, DispEn for the blue color component in ROI 1 (Figure 10A), DispEn for the red color component in ROI 2 (Figure 10B), as well as SumEntrp and entropy for the red color component in ROI 1 (Figure 10C).

For three selected combinations of color components and entropy measures, as well as two selected combinations of color components and entropy-related GLCM texture features, the accuracy of rider:horse bodyweight ratio detection was summarized in Table 1. For both color components and both types of measures/features, a salient observation is that the Se and NPV decreased with higher threshold values (m − SD > mean > m + SD) and the Sp and PPV also increased with higher threshold values (m − SD < mean < m + SD).

For the first threshold (m − SD), Se ranged from 0.92 for DispEn for the red component in ROI 1; through 0.88 for DispEn for the blue component in ROI 1, SumEntrp and entropy for the red component in ROI 1; to 0.79 for DispEn for the red component in ROI 2. In addition, Sp ranged from 0.38 for DispEn for the blue component in ROI 1 and red component in ROI 2; through 0.46 for DispEn for the red component in ROI 1; to 0.63 for SumEntrp and entropy for the red component in ROI 1.

For the second threshold (mean), Se ranged from 0.58 for DispEn for the red component in ROI 1; through to 0.54 for DispEn for the red component in ROI 2; to 0.42 for DispEn for the blue component in ROI 1, SumEntrp and entropy for the red component in ROI 1. In addition, Sp ranged from 0.71 for DispEn for the red component in ROI 2; through 0.92 for DispEn for the red and blue components in ROI 1; to 0.96 for SumEntrp and entropy for the red component in ROI 1.

For the third threshold (m + SD), Se ranged from 0.29 SumEntrp for the red component in ROI 1 to 0.13 for the DispEn for red component in ROI 2. In addition, Sp ranged from 0.96 for DispEn for the red components in ROI 1 to 1.00 for all other concerned entropy measures and features.

## 4. Discussion

In recent research, the IRT imaging of the horses’ bodies was successfully used to differentiate riders with varied bodyweights using conventional [19,20,21,34] and texture analytical [20,34] approaches. However, the accuracy of IRT-based rider:horse bodyweight ratio detection has not been determined yet. In the most recent research, it has been assumed that three entropy-related GLCM texture features, SumEntrp, entropy, and DifEntrp, increased with the rider:horse bodyweight ratio for the red color component of IRT images in the withers area [20]. One may observe that the approaches based on calculating entropy from the matrix obtained from the processing step applied to the image, such as GLCM, represent the disorder of the intermediate matrix rather than the irregularity of the image [63]. In the GLCM approach, the irregularity of pixels in a given window and their likelihood of being similar to the pixels of the next window are investigated. Then, a matrix is created, in which the occurrence of a given pixel in a given image is counted and the entropy is calculated on this basis [55,61]. On the other hand, the entropy measures, such as SampEn, FuzzEn, PermEn, DispEn, and DistEn, are calculated directly on the image; thus, the obtained data are directly related to the irregularity or complexity of the image [45,46,47,48,49]. The entropy measures allow for the estimation of the predictability or uncertainty of spatial patterns in images, where the repeatability of pixel patterns is related to the texture properties of images [56]. Therefore, in the current study, the investigation of thermal images of the horses’ thoracolumbar regions with five detailed entropy measures is fully justified. As Zarychta [43] stated, the usefulness of entropy measures needs to be evaluated in different types of applications [43]; thus, designating their suitability on the equine specimen is also justified.

Following criteria of the applicability of entropy measures for IRT-based rider:horse bodyweight ratio detection, one may notice that none of the combinations for grayscale images differed between the pre-exercise and post-exercise imaging. It means that the transformation of thermal images into color components is required to explore the subtle effort-dependent differences in the irregularity or complexity of this type of images. For the red, green, and blue color components, a similar number of entropy measures passed the first effort-dependent selection criterion, which is in line with the recent results, where the red-green-blue (RGB) color model has already been shown as the most appropriate digital image-processing method applicable in equine IRT and the image texture analysis has been found to be informative in the effort-effect evaluation [64].

Rider:horse bodyweight ratio-dependent differences were noted in the smaller image area for two entropy measures for the red and blue color components, respectively, as well as in the larger image area for the entropy measure for the red and green color component, and two entropy measures for the blue color component. DispEn and DistEn, contrary to SampEn, FuzzEn, and PermEn, passed the second rider:horse bodyweight ratio-dependent criterion. DispEn and DistEn increased with the rider:horse bodyweight ratio, which is in line with the recent suggestions that changes in physiological condition, such as exercise [20,34,64] or pregnancy [44,65], cause a rise in the degree of thermal energy dissipation, and thus entropy of the thermal image texture. The applied entropy measures differ slightly from each other, examining the irregularities in the image by taking into account other elements and returning the results in other intervals [45,46,47,48,49,56]. The SampEn algorithm requires two parameters, m and r, which are not easy to select. The larger the value of m, the greater the chance of no pattern match, whereas choosing an inappropriate value of r leads to an undefined SampEn value [39,56]. SampEn uses a strict binary Heaviside function in determining the degree of similarity between windows. Images with repeated periodic structures (regular patterns) represent a low entropy value, whereas images with non-repeating structures (irregular, unpredictable patterns) represent a high entropy value [56]. Moreover, the SampEn algorithm can give undefined values or unreliable values for small-sized textures [39], which may be the reason for the lack of rider:horse bodyweight ratio-dependent differences for the studied areas of the horses’ back, which are both small. Despite the significant advantages of SampEn, namely simplicity and invariability in rotation [39,56], SampEn could not be selected for further analysis. The FuzzEn algorithm also requires two parameters, n and r, which determine the gradient and the width of the exponential function boundary, respectively, whereas a continuous exponential function is used to determine the degree of similarity [46]. The FuzzEn, similarly to SampEn, is invariant to rotation and is invariant to translation; however, the sensitivity of measure selection is lower for FuzzEn than SampEn [46]. The low sensitivity may be the reason for the lack of rider:horse bodyweight ratio-dependent differences, and thus lack of selection for further analysis. PermEn examines the repeatability of pixel patterns, which is related to the texture properties of images. If the image pixels are highly disordered, the value of permutation entropy is close to unity, whereas if the pixels occur in the same order, the value of PermEn is close to zero [58]. The average PermEn value of periodic, highly ordered textures is lower than for synthesized textures, which makes it possible to distinguish periodic from synthesized textures [58]. Unfortunately, in the PermEn algorithm, the equal or repeated values can introduce errors in the estimation of the probability distribution of the order pattern during the calculation [66], which may result in no differences in the rider:horse bodyweight ratio. The DistEn algorithm eliminates the drawbacks of SampEn of being not very sensitive to the values of the parameters m and r [49]. Moreover, the DistEn algorithm eliminates undefined values for small image sizes, which the SampEn, FuzzyEn, or PermEn algorithms could not handle [56]. If all the possible scatter patterns of the image have equal probability values, DispEn reaches the highest value, indicating that the image is irregular. If the image is regular, DispEn reaches the lowest value [48,57]. The DistEn, similarly to SampEn and FuzzEn, is invariant to rotation [49,56], which together with DistEn effectiveness in assessing small images may make DistEn useful for further analysis for both smaller and larger image areas. The DispEn algorithm requires two parameters as well, m and c, which remain the least sensitive to rotation, translation and image size compared to SampEn, FuzzEn, PermEn, and DistEn [57]. Moreover, for DispEn, the greatest rider:horse bodyweight ratio-dependent differences were demonstrated, which makes DispEn one of the most promising measures selected for further analysis for both smaller and larger image areas. Therefore, one may note that the thermal images obtained after exercise with a rider with a high rider:horse bodyweight ratio showed a more irregular and complex texture than thermal images obtained from a low rider:horse bodyweight ratio group.

The results of the current study show that DispEn and DistEn for three color components in the RGB color model are useful to indicate differences between the light and heavy rider groups. However, their applicability required further consideration against the recent results that represented the entropy-related GLCM texture features [20]. One may observe that in the smaller image area, the slopes of the linear regression equations for DispEn for the red and blue color components were the most similar to the slope of the linear regression equations for SumEntrp and Entropy for the red color component and achieved the highest slope values. Significant similarities were also observed for DispEn for the red color component in the larger image area. This means that entropy measures selected based on the currently used specific criteria represent the greatest changes in thermal image texture between the light and heavy rider groups, which are consistent with the previous observations [20], and therefore repeatable in a more extensive experimental model. However, the greatest changes in the thermal image texture were observed only for red and blue color components. On the thermal images, the temperatures are coded with respective image colors; the red component reflects the high temperature, whereas the blue one reflects the low temperature [67]. Therefore, the greatest changes noted currently in the red and blue color components are in line with conventional thermal results, where two temperature measures, the minimal and maximal, differed both between effort states [33,64] and different rider bodyweights [20,21,34]. Those differences reflect the thermal energy emission increase with the increase in metabolic energy produced by loaded muscle units [15,16,67,68]. Interestingly, as the DispEn algorithm returned entropy measures that were not very sensitive to rotation, translation, or image size [57], DispEn may be applicable in smaller or larger image areas, unlike the entropy-related GLCM texture features [20]. This is probably because one of the GLCM method imperfections is the nonsystematic coverage and poor presentation of image scales and directions [63]. Thus, the applicability of DispEn seems to be wider than the previously used entropy features, which was supported by the estimation of the accuracy of rider:horse bodyweight ratio detection.

One may observe that the sensitivity and specificity of the light and heavy rider groups’ differentiation were the highest precisely for DispEn for the red color component in the smaller image area. The accuracy of distinguishing between the light and heavy rider groups was similar for DispEn for the blue color component and previously used entropy features for the red color component in the same size of image area. However, the studied accuracy decreased with the increasing size of image area, being the lowest for DispEn for the red color component in the larger image area. As the GLCM method is best suited to the detection of small lesions in low-resolution images [63], the new application of DispEn presented here may be used in equine medicine and management.

Two-dimensional entropy measures are simple to implement and are based on well-developed theoretical aspects [56,57]; however, they are not commercially available, contrary to the GLCM approach. The GLCM features are returned using the QMazda Software [61], which is one of the easiest standalone softwares for image texture analysis. The graphical user interface is simple and clear, and one can quickly run several analyses and generate fully customized results that can be exported as raw data or plots [61]. The accessibility of the QMazda Software is an advantage for large population studies, increasing the availability of texture analysis methods in research studies. Two-dimensional entropy measures represent relatively recent computational methods, whose commercial use requires programming and time-consuming calculations [45,46,47,48,49]. However, for research purposes, this inconvenience is disproportionately small compared to the benefits of obtaining entropy data, which are directly related to the irregularity or complexity of the image. It can be concluded that the simplification of the two-dimensional entropy measures calculation, by creating commercially available, easy-to-use software, providing real-time or near real-time feedback, will popularize the entropy evaluation from thermal images, which will be beneficial in various areas of equine medicine and management, such as equids-specific superficial body temperature pattern determination [22], evaluation of the load on the horse’s back [20,21,34], detection of exercise-induced changes [33,64], horse-rider-matching [19], saddle fitting [35], and pregnancy recognition [44,65]. One may summarize that the presented data demonstrate the preliminary use of entropy measures to select the most informative measures and color components, and the accuracy of rider: horse bodyweight ratio detection; therefore, further research is required on different horse breeds, under varying environmental conditions in different countries, to confirm the practical suitability of this infrared-based sensing for monitoring equine physiology.

## 5. Conclusions

Thermal images obtained from the equine thoracolumbar region among riders that represent different rider:horse bodyweight ratios can be successfully investigated by five entropy measures after conversion to three basic color components. The evidence of higher irregularity and complexity of thermal image texture was noted for red and blue color components using two (DispEn and DistEn) out of five explored entropy measures, when horses worked under riders with a high rider:horse bodyweight ratio. An application of DispEn to the red color component makes it possible to identify the light and heavy rider groups with higher accuracy than the previously used entropy-related GLCM texture features. However, advancement in the available software for two-dimensional entropy measures is required to facilitate the use of this promising measurement method in various areas of equine medicine and management.

## Figures and Tables

**Table 1:** The sensitivity (Se), specificity (Sp), positive predictive value (PPV), and negative predictive value (NPV) of distinguishing of light (L) and heavy (H) rider groups based on the selected entropy measures (DispEn—two-dimensional dispersion entropy, DistEn—two-dimensional distribution entropy) and the selected gray-level run-length matrix features (SumEntrp—summation entropy, entropy) for selected color components (red, blue) and areas (ROI 1—the withers area, ROI 2—the thoracic spine area). Three thresholds (mean − SD (m − SD), mean, mean + SD (m + SD)) were used.

Measures	DispEn			DispEn			DispEn			SumEntrp			Entropy		
Component	Red			Blue			Red			Red			Red		
Area	ROI 1			ROI 1			ROI 2			ROI 1			ROI 1		
Threshold	m − SD	Mean	m + SD	m − SD	Mean	m + SD	m − SD	Mean	m + SD	m − SD	Mean	m + SD	m − SD	Mean	m + SD
Se	0.92	0.58	0.17	0.88	0.42	0.25	0.79	0.54	0.13	0.88	0.42	0.29	0.88	0.42	0.25
Sp	0.46	0.92	0.96	0.38	0.92	1.00	0.38	0.71	1.00	0.63	0.96	1.00	0.63	0.96	1.00
PPV	0.63	0.80	0.88	0.58	0.83	1.00	0.56	0.65	1.00	0.70	0.91	1.00	0.70	0.91	1.00
NPV	0.85	0.69	0.53	0.75	0.61	0.57	0.64	0.61	0.53	0.83	0.62	0.59	0.83	0.62	0.57

## Data Availability

The data presented in this study are available upon request from the corresponding author.

## Supplementary Materials

The following are available online at https://www.mdpi.com/1424-8220/22/16/6052/s1, Table S1: Details of horses and riders participating in the study. Riders represented the light (L), moderate (M), and heavy (H) riders groups, Table S2: Details of the rider:horse bodyweight ratio of pairs participating in the study. Riders represented the light (L), moderate (M), and heavy (H) rider groups, Table S3: The withers area (ROI 1). Values (mean ± SD) of entropy measures for grayscale images in light (L), moderate (M), and heavy (H) groups, Table S4: The thoracic spine area (ROI 2). Values (mean ± SD) of entropy measures for grayscale images in light (L), moderate (M), and heavy (H) groups, Table S5: The withers area (ROI 1). Values (mean ± SD) of entropy measures for red color component in light (L), moderate (M), and heavy (H) groups. Table S6: The thoracic spine area (ROI 2). Values (mean ± SD) of entropy measures red color component in light (L), moderate (M), and heavy (H) groups, Table S7: The withers area (ROI 1). Values (mean ± SD) of entropy measures for green color component in light (L), moderate (M), and heavy (H) groups, Table S8: The thoracic spine area (ROI 2). Values (mean ± SD) of entropy measures green color component in light (L), moderate (M), and heavy (H) groups, Table S9: The withers area (ROI 1). Values (mean ± SD) of entropy measures for blue color component in light (L), moderate (M), and heavy (H) groups, Table S10: The thoracic spine area (ROI 2)**.** Values (mean ± SD) of entropy measures blue color component in light (L), moderate (M), and heavy (H) groups.
