# Supplementary material for: Application of the Two-Dimensional Entropy Measures in the Infrared Thermography-Based Detection of Rider: Horse Bodyweight Ratio in Horseback Riding
Source: Sensors (Basel). 2022 Aug 13;22(16):6052. doi: 10.3390/s22166052 (PMC9414866; doi:10.3390/s22166052)
Supplement: Supplementary file 1 [file sensors-22-06052-s001.zip › sensors-1857631-supplementary.pdf]

Supplementary Data for

Application of the Two-Dimensional Entropy Measures in the Infrared Thermography-Based Detection of Rider:Horse Bodyweight Ratio in Horseback Riding

Małgorzata Domino [1,*], Marta Borowska [2], Łukasz Zdrojkowski [1], Tomasz Jasiński [1], Urszula Sikorska [3] , Michał Skibniewski [4] and Małgorzata Maśko [3,*]

[1] Department of Large Animal Diseases and Clinic, Institute of Veterinary Medicine, Warsaw University of Life Sciences, 02-787 Warsaw, Poland

[2] Institute of Biomedical Engineering, Faculty of Mechanical Engineering, Białystok University of Technology, 15-351 Bialystok, Poland

[3] Department of Animal Breeding, Institute of Animal Science, Warsaw University of Life Sciences, 02-787 Warsaw, Poland

[4] Department of Morphological Sciences, Institute of Veterinary Medicine, Warsaw University of Life Sciences, 02-776 Warsaw, Poland

* Correspondence: malgorzata_domino@sggw.edu.pl (M.D.); malgorzata_masko@sggw.edu.pl (M.M.)

Table S1. Details of horses and riders participating in the study. Riders represented the light (L), moderate (M), and heavy (H) riders groups.

Rider groups	Horse				Horse				Rider			
	**Sign**	**BW**	**High**	**SW**	**Sign**	**BW**	**High**	**SW**	**Sign**	**BW**	**High**	**BMI**
L	**1**	585	166	4.1	**7**	560	162	4.2	**A**	58	159	22.9
	**2**	550	154	4.5	**8**	545	157	4.2	**B**	60	160	23.4
M	**3**	580	158	4.2	**9**	570	158	4.4	**C**	77	172	26.0
	**4**	575	164	4.5	**10**	580	162	4.4	**D**	75	164	27.9
H	**5**	560	160	4.1	**11**	565	160	4.2	**E**	91	172	30.8
	**6**	550	156	4.4	**12**	580	166	4.2	**F**	92	174	30.4

SW- saddle's weight [kg]; BW- bodyweight [kg]; height [cm]; BMI- body mass index [kg/m^2].

Table S2. Details of the rider:horse bodyweight ratio of pairs participating in the study. Riders represented the light (L), moderate (M), and heavy (H) riders groups.

Rider groups	Horse/ Rider	Rider: Horse Bodyweight Ratio											
		**1**	**2**	**3**	**4**	**5**	**6**	**7**	**8**	**9**	**10**	**11**	**12**
L	**A**	10.6	11.4	10.7	10.9	11.1	11.3	11.1	11.4	10.9	10.8	11.0	10.7
	**B**	11.0	11.7	11.1	11.2	11.4	11.7	11.5	11.8	11.3	11.1	11.4	11.1
M	**C**	13.9	14.8	14.0	14.2	14.5	14.8	14.5	14.9	14.3	14.0	14.4	14.0
	**D**	13.5	14.5	13.7	13.8	14.1	14.4	14.1	14.5	13.9	13.7	14.0	13.7
H	**E**	16.3	17.4	16.4	16.6	17.0	17.3	17.0	17.5	16.7	16.4	16.8	16.4
	**F**	16.4	17.5	16.6	16.8	17.2	17.5	17.2	17.7	16.9	16.6	17.0	16.6

Horse - from 1 to 12; Rider - from A to F.

Table S3. The withers area (ROI 1)**.** Values (mean ± SD) of entropy measures for grayscale images in light (L), moderate (M), and heavy (H) groups. The pre-exercise (pre-ex) and post exercise (post-ex) data series were compared. When features differed significantly ($p < 0.05$) for all three groups (L, M, H), the values were marked in bold.

Groups		SampEn	FuzzEn	PermEn	DispEn	DistEn
	pre-ex	1.14±0.39	1.77±0.45	1.20±0.04	7.01±0.51	0.93±0.06
L	post-ex	1.06±0.25	1.63±0.29	1.19±0.04	7.00±0.41	0.93±0.04
	p	0.500	0.262	0.509	0.622	0.812
	pre-ex	1.04±0.41	1.71±0.50	1.20±0.03	7.08±0.51	0.92±0.05
M	post-ex	1.21±0.38	1.83±0.45	1.20±0.04	7.18±0.37	0.92±0.03
	p	0.065	0.375	0.877	0.318	> 0.999
	pre-ex	1.20±0.33	1.92±0.43	1.21±0.02	7.23±0.36	0.93±0.05
H	post-ex	1.22±0.43	1.78±0.49	1.21±0.03	7.22±0.45	0.94±0.03
	p	0.822	0.170	0.375	0.923	0.855

ROI - regions of interest. SampEn - two-dimensional sample entropy, FuzzEn - two-dimensional fuzzy entropy, PermEn - two-dimensional permutation entropy, DispEn - two-dimensional dispersion entropy, DistEn - two-dimensional distribution entropy. p - the level of marginal significance; SD - standard deviation.

Table S4. The thoracic spine area (ROI 2)**.** Values (mean ± SD) of entropy measures for grayscale images in light (L), moderate (M), and heavy (H) groups. The pre-exercise (pre-ex) and post exercise (post-ex) data series were compared. When features differed significantly ($p < 0.05$) for all three groups (L, M, H), the values were marked in bold.

Groups		SampEn	FuzzEn	PermEn	DispEn	DistEn
	pre-ex	**0.84±0.33**	**1.46±0.35**	**1.15±0.06**	6.80±0.54	**0.95±0.04**
L	post-ex	**1.21±0.35**	**1.75±0.41**	**1.19±0.03**	6.96±0.43	**0.93±0.04**
	p	**0.001**	**0.016**	**0.001**	0.164	**0.046**
	pre-ex	**0.81±0.33**	**1.41±0.40**	1.15±0.05	6.82±0.47	0.94±0.04
M	post-ex	**1.10±0.40**	**1.61±0.35**	1.18±0.05	6.87±0.57	0.93±0.04
	p	**0.003**	**0.049**	0.051	0.944	0.114
	pre-ex	1.07±0.48	1.67±0.41	1.18±0.04	7.03±0.43	0.92±0.06
H	post-ex	1.17±0.47	1.72±0.51	1.17±0.08	6.92±0.93	0.90±0.08
	p	0.456	0.838	0.456	> 0.999	0.143

ROI - regions of interest. SampEn - two-dimensional sample entropy, FuzzEn - two-dimensional fuzzy entropy, PermEn - two-dimensional permutation entropy, DispEn - two-dimensional dispersion entropy, DistEn - two-dimensional distribution entropy. p - the level of marginal significance; SD - standard deviation.

Table S5. The withers area (ROI 1)**.** Values (mean ± SD) of entropy measures for red color component in light (L), moderate (M), and heavy (H) groups. The pre-exercise (pre-ex) and post exercise (post-ex) data series were compared. When features differed significantly ($p < 0.05$) for all three groups (L, M, H), the values were marked in bold.

Groups		SampEn	FuzzEn	PermEn	DispEn	DistEn
	pre-ex	0.61±0.35	1.38±0.60	1.17±0.05	**7.25±0.39**	**0.83±0.15**
L	post-ex	0.94±0.74	1.15±0.29	1.19±0.04	**6.57±0.47**	**0.50±0.14**
	p	0.089	0.218	0.345	**< 0.0001**	**< 0.0001**
	pre-ex	0.55±0.31	**1.34±0.58**	1.17±0.05	**7.34±0.42**	**0.85±0.14**
M	post-ex	0.55±0.42	**0.99±0.24**	1.19±0.04	**6.87±0.68**	**0.59±0.21**
	p	0.584	**0.020**	0.152	**0.005**	**< 0.0001**
	pre-ex	0.54±0.27	**1.39±0.50**	**1.15±0.04**	**7.45±0.35**	**0.90±0.09**
H	post-ex	0.59±0.31	**1.14±0.27**	**1.22±0.08**	**7.11±0.55**	**0.64±0.23**
	p	0.550	**0.034**	**< 0.0001**	**0.007**	**< 0.0001**

ROI - regions of interest. SampEn - two-dimensional sample entropy, FuzzEn - two-dimensional fuzzy entropy, PermEn - two-dimensional permutation entropy, DispEn - two-dimensional dispersion entropy, DistEn - two-dimensional distribution entropy. p - the level of marginal significance; SD - standard deviation.

Table S6. The thoracic spine area (ROI 2)**.** Values (mean ± SD) of entropy measures red color component in light (L), moderate (M), and heavy (H) groups. The pre-exercise (pre-ex) and post exercise (post-ex) data series were compared. When features differed significantly (p < 0.05) for all three groups (L, M, H), the values were marked in bold.

Groups		SampEn	FuzzEn	PermEn	DispEn	DistEn
	pre-ex	**0.82±0.83**	**0.91±0.37**	**1.13±0.06**	**6.05±0.69**	**0.81±0.14**
L	post-ex	**1.98±1.60**	**1.52±0.32**	**1.18±0.04**	**6.34±0.71**	**0.63±0.14**
	p	**0.001**	**< 0.0001**	**0.004**	**0.037**	**0.0001**
	pre-ex	**0.61±0.69**	**0.84±0.36**	**1.13±0.06**	**6.18±0.60**	**0.82±0.14**
M	post-ex	**1.16±0.89**	**1.42±0.36**	**1.17±0.06**	**6.68±0.93**	**0.66±0.14**
	p	**0.001**	**< 0.0001**	**0.033**	**0.025**	**0.0001**
	pre-ex	**0.56±0.67**	**0.86±0.30**	1.15±0.06	**6.36±0.76**	**0.87±0.12**
H	post-ex	**1.11±1.08**	**1.41±0.40**	1.18±0.05	**6.91±0.99**	**0.67±0.19**
	p	**0.003**	**< 0.0001**	0.160	**0.018**	**0.002**

ROI - regions of interest. SampEn - two-dimensional sample entropy, FuzzEn - two-dimensional fuzzy entropy, PermEn - two-dimensional permutation entropy, DispEn - two-dimensional dispersion entropy, DistEn - two-dimensional distribution entropy. p - the level of marginal significance; SD - standard deviation.

Table S7. The withers area (ROI 1)**.** Values (mean ± SD) of entropy measures for green color component in light (L), moderate (M), and heavy (H) groups. The pre-exercise (pre-ex) and post exercise (post-ex) data series were compared. When features differed significantly (p < 0.05) for all three groups (L, M, H), the values were marked in bold.

Groups		SampEn	FuzzEn	PermEn	DispEn	DistEn
	pre-ex	**1.13±0.49**	2.22±0.61	**1.16±0.04**	**7.45±0.26**	**0.88±0.07**
L	post-ex	**0.89±0.29**	1.96±0.45	**1.14±0.03**	**7.71±0.25**	**0.98±0.02**
	p	**0.038**	0.106	**0.025**	**0.0002**	**< 0.0001**
	pre-ex	**1.05±0.32**	2.16±0.46	**1.17±0.04**	**7.52±0.25**	**0.88±0.08**
M	post-ex	**0.82±0.25**	1.96±0.47	**1.15±0.03**	**7.74±0.19**	**0.97±0.02**
	p	**0.004**	0.065	**0.039**	**0.0004**	**< 0.0001**
	pre-ex	1.06±0.43	2.13±0.59	**1.17±0.04**	**7.48±0.27**	**0.85±0.06**
H	post-ex	0.93±0.34	2.06±0.54	**1.14±0.02**	**7.76±0.33**	**0.97±0.03**
	p	0.240	0.650	**0.0008**	**0.003**	**< 0.0001**

ROI - regions of interest. SampEn - two-dimensional sample entropy, FuzzEn - two-dimensional fuzzy entropy, PermEn - two-dimensional permutation entropy, DispEn - two-dimensional dispersion entropy, DistEn - two-dimensional distribution entropy. p - the level of marginal significance; SD - standard deviation.

Table S8. The thoracic spine area (ROI 2)**.** Values (mean ± SD) of entropy measures green color component in light (L), moderate (M), and heavy (H) groups. The pre-exercise (pre-ex) and post exercise (post-ex) data series were compared. When features differed significantly (p < 0.05) for all three groups (L, M, H), the values were marked in bold.

Groups		SampEn	FuzzEn	PermEn	DispEn	DistEn
	pre-ex	**0.73±0.32**	**1.79±0.54**	1.12±0.04	**7.28±0.46**	**0.92±0.05**
L	post-ex	**1.45±0.41**	**2.86±0.61**	1.10±0.04	**7.69±0.22**	**0.85±0.07**
	p	**< 0.0001**	**< 0.0001**	0.088	**0.0007**	**0.0004**
	pre-ex	**0.76±0.35**	**1.84±0.68**	1.13±0.04	**7.38±0.39**	**0.95±0.05**
M	post-ex	**1.07±0.41**	**2.23±0.63**	1.13±0.05	**7.70±0.34**	**0.89±0.07**
	p	**0.008**	**0.044**	0.793	**0.004**	**0.004**
	pre-ex	0.91±0.45	2.07±0.62	1.12±0.05	**7.10±0.53**	**0.97±0.03**
H	post-ex	1.12±0.63	2.27±0.99	1.13±0.04	**7.71±0.53**	**0.92±0.06**
	p	0.303	0.812	0.353	**0.0002**	**0.0002**

ROI - regions of interest. SampEn - two-dimensional sample entropy, FuzzEn - two-dimensional fuzzy entropy, PermEn - two-dimensional permutation entropy, DispEn - two-dimensional dispersion entropy, DistEn - two-dimensional distribution entropy. p - the level of marginal significance; SD - standard deviation.

Table S9. The withers area (ROI 1)**.** Values (mean ± SD) of entropy measures for blue color component in light (L), moderate (M), and heavy (H) groups. The pre-exercise (pre-ex) and post exercise (post-ex) data series were compared. When features differed significantly ($p < 0.05$) for all three groups (L, M, H), the values were marked in bold.

Groups		SampEn	FuzzEn	PermEn	DispEn	DistEn
L	pre-ex	**1.09±0.71**	**1.71±0.51**	1.19±0.03	**7.77±0.22**	**0.86±0.09**
	post-ex	**1.54±0.45**	**2.41±0.43**	1.18±0.02	**7.02±0.26**	**0.72±0.11**
	p	**0.013**	**< 0.0001**	0.050	**< 0.0001**	**0.021**
M	pre-ex	**0.83±0.40**	**1.51±0.41**	**1.20±0.02**	**7.65±0.28**	**0.87±0.08**
	post-ex	**1.17±0.41**	**2.04±0.45**	**1.18±0.02**	**7.21±0.35**	**0.80±0.11**
	p	**0.007**	**< 0.0001**	**0.045**	**< 0.0001**	**< 0.0001**
H	pre-ex	**0.70±0.31**	**1.43±0.30**	1.19±0.03	**7.65±0.33**	**0.89±0.09**
	post-ex	**1.10±0.35**	**1.96±0.40**	1.19±0.02	**7.37±0.35**	**0.84±0.09**
	p	**0.0003**	**< 0.0001**	0.288	**0.010**	**0.032**

ROI - regions of interest. SampEn - two-dimensional sample entropy, FuzzEn - two-dimensional fuzzy entropy, PermEn - two-dimensional permutation entropy, DispEn - two-dimensional dispersion entropy, DistEn - two-dimensional distribution entropy. p - the level of marginal significance; SD - standard deviation.

Table S10. The thoracic spine area (ROI 2)**.** Values (mean ± SD) of entropy measures blue color component in light (L), moderate (M), and heavy (H) groups. The pre-exercise (pre-ex) and post exercise (post-ex) data series were compared. When features differed significantly ($p < 0.05$) for all three groups (L, M, H), the values were marked in bold.

Groups		SampEn	FuzzEn	PermEn	DispEn	DistEn
L	pre-ex	**1.33±0.52**	**2.08±0.44**	1.18±0.04	**7.89±0.20**	**0.84±0.09**
	post-ex	**1.78±0.49**	**2.34±0.49**	1.19±0.02	**7.45±0.30**	**0.71±0.11**
	p	**< 0.0001**	**0.004**	0.327	**< 0.0001**	**< 0.0001**
M	pre-ex	1.18±0.46	1.97±0.41	1.19±0.02	**7.82±0.13**	**0.86±0.07**
	post-ex	1.39±0.49	2.04±0.46	1.19±0.04	**7.57±0.26**	**0.77±0.11**
	p	0.128	0.527	0.812	**0.0001**	**0.0008**
H	pre-ex	1.26±0.50	2.03±0.46	1.19±0.03	**7.84±0.12**	**0.85±0.08**
	post-ex	1.38±0.80	1.97±0.65	1.19±0.02	**7.66±0.27**	**0.81±0.07**
	p	0.790	0.591	0.931	**0.016**	**0.026**

ROI - regions of interest. SampEn - two-dimensional sample entropy, FuzzEn - two-dimensional fuzzy entropy, PermEn - two-dimensional permutation entropy, DispEn - two-dimensional dispersion entropy, DistEn - two-dimensional distribution entropy. p - the level of marginal significance; SD - standard deviation.
